# Effect of an intensive 3-day social cognitive treatment (can do treatment) on control self-efficacy in patients with relapsing remitting multiple sclerosis and low disability: A single-centre randomized controlled trial

**DOI:** 10.1371/journal.pone.0223482

**Published:** 2019-10-10

**Authors:** Peter Joseph Jongen, Ghislaine A. van Mastrigt, Marco Heerings, Leo H. Visser, Rob P. Ruimschotel, Astrid Hussaarts, Lotte Duyverman, Joyce Valkenburg-Vissers, Job Cornelissen, Michel Bos, Maarten van Droffelaar, Rogier Donders

**Affiliations:** 1 Department of Community & Occupational Medicine, University Medical Centre Groningen, University of Groningen, Groningen, the Netherlands; 2 MS4 Research Institute, Nijmegen, the Netherlands; 3 Department of Health Services Research, CAPHRI Care and Public Health Research Institute, Maastricht University, Maastricht, the Netherlands; 4 National Multiple Sclerosis Foundation, Rotterdam, the Netherlands; 5 Department of Neurology, St. Elisabeth Hospital, Tilburg, the Netherlands; 6 University of Humanistic Studies, Utrecht, the Netherlands; 7 Medical Psychiatric Centre PsyToBe, Rotterdam, the Netherlands; 8 Fysiotherapie Maaspoort, 's-Hertogenbosch, the Netherlands; 9 Dansjobs, Landsmeer, the Netherlands; 10 Department of Neurology, St. Anna Hospital, Geldrop, the Netherlands; 11 De Firma Drof, Utrecht, the Netherlands; 12 Department for Health Evidence, Radboud University Medical Center, Nijmegen, the Netherlands; University Medical Center Gottingen, GERMANY

## Abstract

In patients with chronic disorders, control self-efficacy is the confidence with managing symptoms and coping with the demands of illness. Can do treatment (CDT) is an intensive, 3-day, social cognitive theory-based, multidisciplinary treatment that focuses on identification of stressors, goal setting, exploration of boundaries, and establishment of new boundaries. An uncontrolled study showed that patients with relapsing remitting multiple sclerosis (RRMS) and low-disability had improved control self-efficacy six months after CDT. Hence, in a 6-month, single-centre, randomized (1:1), unmasked, controlled trial in RRMS patients with Expanded Disability Status Scale (EDSS) score ≤4.0, we compared CDT with no intervention and the option to receive CDT after completion of study participation. Follow-up assessments were at one, three and six months. Primary endpoint was control self-efficacy (Multiple Sclerosis Self-Efficacy Scale Control [MSSES-C] (minimum 90, maximum 900) at six months. Secondary endpoints were functional self-efficacy (MSSES-F), participation and autonomy (Impact on Participation and Autonomy questionnaire [IPA]), health-related quality of life (MS Quality of Life-54 Items questionnaire [MSQoL-54]), anxiety, depression (Hospital Anxiety and Depression Scale [HADS]) and coping skills (Utrecht Coping List [UCL]) at six months. Tertiary endpoint was care-related strain on support partners (Caregiver Strain Index) at six months. Of the 158 patients that were included, 79 were assigned to CDT and 79 to the control group. Two CDT patients discontinued treatment prematurely. Sixty-one (77%) control patients chose to receive CDT after study participation. Intention-to-treat ANCOVA analyses were performed with follow-up values as dependent, and condition, baseline values, disease duration and gender as independent variables. The mean (standard deviation [SD]) MSSES-C score in the CDT group vs. control group at baseline was 468 (162) vs. 477 (136), and at six months 578 (166) vs. 540 (135) (p = 0.100). Secondary and tertiary endpoints did not differ between groups, except for the UCL palliative reaction score being slightly higher in the CDT group (p = 0.039). On post hoc analyses the MSSES-C score at one and three months was higher in the CDT vs. control group: 597 (114) vs. 491 (131) (p<0.0001) and 561 (160) vs. 514 (143) (p = 0.018), respectively; and at one month the MSSES-F, IPA Limitations, HADS Anxiety and Depression, and MSQoL-54 Mental and Physical scores were also in favour of the CDT group. We conclude that in low-disability RRMS patients, the intensive 3-day social cognitive theory-based CDT did not improve control self-efficacy at six months follow-up compared to waitlist controls. The absence of a between-group difference at six months relates to a gradual improvement in the control group. In all, this social cognitive theory-based approach for improving self-efficacy needs further investigation before being broadly applied in RRMS patients.

## Introduction

Multiple sclerosis (MS) is a chronic inflammatory, demyelinating and degenerative disease of the central nervous system. Intermittent or continuous disease activity frequently results in a stepwise or slow increase in disabilities. The disease course is largely unpredictable, as is the response to disease-modifying drug treatment. It is therefore unsurprising that, due to experiences of disappearing abilities and the uncertainty regarding their future, MS patients may lose their self-confidence.

Self-efficacy is a psychological concept that refers to the degree to which a person is confident to be able to complete tasks and reach goals in specific situations [1]. It is a core component in social cognitive theory, according to which psychosocial functioning is determined by reciprocal interactions between personal factors, behavior and the environment. Self-efficacy is a strong predictor of health behavior, and it can be instrumental in modulating the experience of chronic illness. In MS patients low self-efficacy is associated with lower health-related quality of life (HRQoL) [2], less mood control [3], less social activity [3] and less physical activity [4]. In recent years various interventions have been developed aiming to increase self-efficacy in patients with MS. However, evidence form randomized controlled trials (RCT) for the effectiveness of these approaches is scarce and variable [5–8].

We previously reported findings from an observational study on self-efficacy in MS patients after can do treatment (CDT), an intensive 3-day multidisciplinary social cognitive theory-based intervention [9]. Six months after CDT, patients with relapsing remitting (RR) MS and those with low disability (Expanded Disability Status Scale [EDSS] score <4.0) showed an improvement of control self-efficacy, i.e. improved confidence in managing symptoms and coping with the demands of illness [9].

In view of these findings we performed in low-disability RRMS patients a 6-month, single-centre, unmasked RCT to investigate the effect of CDT on control self-efficacy, with control patients receiving no CDT and having the option to receive CDT after completion of study participation. The primary outcome was the between-group difference at six months. Our hypothesis was that control self-efficacy would be higher in the CDT than in the control group.

## Materials and methods

### Can do treatment

#### Concept

CDT is a social cognitive theory-based intervention aiming to uncover and promote existing capabilities, with the notion ‘stressor’ as central concept [9]. CDT is based on the identification and reduction of stressors; client-centeredness; potential inclusion of partner or another significant informal caregiver; group sessions; and self-reliance, autonomy and acceptance as central themes [9]. CDT focuses on the exploration of those stressors that confine patients to their physical, psychological or social roles regarding their disease and limitations; reduces relevant stressors; explores and pushes personal boundaries; and creates new personal boundaries by making optimal use of the existing potential [9]. To place the individual’s capabilities in a realistic framework, CDT’s central themes are ‘Can’, ‘Will’, ‘Choose’, ‘Open up to others’, and ‘Do’. CDT hypothesizes that by exploring their boundaries patients become more aware of their faculties, and that the higher awareness of potentials leads to self-management [9].

#### Components

CDT’s components are large group sessions, small group sessions, consultations (carrousel), a theatre evening, and start of the day with a joint activity (optional) [9].

Large group sessions include plenary sessions–where participants make optimal use of their existing potentials, learn how to support and encourage other participants, and experiment how to give the required feedback to the multidisciplinary team—, and group sessions in which half of the participants take part–where they examine and identify which stressors have to be addressed most, and formulate one or two realizable individual aims [9].

After having identified and formulated individual stressors and aims, participants sign in for one or more group consultations, during which they verify whether their aims are realizable by asking the members of the multidisciplinary team for aim-related medical information [9].

Small group sessions form the actual training [9]. Depending on their individual goals the participants sign up for the training groups ‘Body’, ‘Feeling’ or ‘Life’, to work out their aims and to experiment whether they can reduce their stressors. Body sessions focus on the exploration of physical capabilities and are coached by a physiotherapist. Feeling sessions focus on the exploration of the emotional potential and are coached by a psychiatrist and a psychiatric nurse. Life sessions focus on the exploration of capabilities relating to daily living with MS and are coached by a neurologist, a registered nurse specialized in MS, and a person with MS. In addition, there are relaxation sessions for those who have difficulties in experiencing their body: dance sessions focus on body experience and relaxation, and make participants aware of the relationship between physical sensations and their emotions and feelings, whereas physical sessions focus on relaxation through physical strain. Participants autonomously chose between the various small group sessions without being influenced by team members [9].

On the informal theatre evening the participants practice to change roles and to show their potentials by openly experimenting. They do their best to perform before each other and the team. The jointly created evening performance increases the cohesion within the group and learns participants to find an equilibrium between consuming and action [9].

During an optional joint activity (walk in the woods) at the start of the day the participants experiment with physical challenges and with the management of their energy [9].

#### Multidisciplinary team

The multidisciplinary team included a psychiatrist, psychiatric nurse, neurologist, registered nurse specialized in MS, physiotherapist, dance therapist, and a person with MS [9]. All professional team members were formally trained in their respective disciplines and all members were experienced in the field of MS. The person with MS had experience with chronic symptoms and multiple disease-modifying treatments. As the team members were also involved in the preceding observational study—except for the dance therapist -, the team was experienced in providing CDT [9]. Before starting the first CDT of the observational study, team members held two plenary sessions, in which the psychiatrist and psychiatric nurse informed the other members about this social cognitive theory-based approach, and during which the team became familiar with the concept, and each team member’s specific contribution to CDT.

The members respected and understood the participants’ individual qualities and differences, and they stimulated, defied and confronted them to explore and push their boundaries [9]. Apart from the consultations the team kept to coaching, stimulating and activating the participants. By participating in the large group sessions team members became familiar with the participants’ individual stressors and goals. During the consultations they had a professional and informative role. In the small group sessions every team member focused on its own area of interest. During a tip time at the end of each day the team members evaluated the sessions, informed each other on the participants’ progresses and obstacles, discussed whether the participants made optimal use of their opportunities, monitored to what extent the personal goals were being attained, and noted adverse events [9]. At the end of the intervention any premature discontinuations were documented.

### Study design and participants

We did a single-centre, randomized (1:1), unmasked, parallel-group, controlled trial in the Netherlands. The first version of the study protocol (September 2012) has been extended with an economic evaluation, the Utrecht Coping List (UCL), and information about financial aspects and randomization procedure (amended version March 2013). The ethics approval was obtained on February 11, 2013 from the ethical committee *Medisch Ethische Toetsing Onderzoek Patiënten en Proefpersonen*, Tilburg, the Netherlands. *Medische Ethische Toetsing Commissie* (METC) number 499; NL number: NL4220502812. Dutch Trial Registry: Trial NL5158 (NTR5298) (https://www.trialregister.nl/trial/5158). Due to a miscommunication between the MS4 Research Institute, Nijmegen, the Netherlands, and the National Multiple Sclerosis Foundation (NMSF), Rotterdam, the Netherlands, the trial was registered after the inclusion had started, namely July 10, 2015. The authors confirm that all ongoing and related trials for this intervention are registered. The study protocol has been published May 28, 2016, including an adapted statistical section, as we considered the 6-month outcome more informative and clinically relevant than the mean of the 3-month and 6-month values [10] (https://bmcneurol.biomedcentral.com/articles/10.1186/s12883-016-0593-4).

The study population was low-disability RRMS patients with or without support partner. Eligibility criteria for patients: diagnosis RRMS since at least one year, EDSS score ≤4.0, no symptoms suggestive of a relapse, no relapse in the preceding four weeks, willing and capable of participating in the investigations, and having access to the internet for performing the web-based assessments. Eligibility criteria for support partners: willing and capable of participating in the investigations, and having access to the internet.

Participants were recruited from all over the Netherlands by the patient organisation NMSF, Rotterdam, the Netherlands. Interested patients were contacted by a registered nurse experienced in MS (MH) by phone to check diagnosis and eligibility criteria. The MS diagnosis was not confirmed by a neurologist. The criterion of EDSS ≤4.0 was checked by assessing whether the patient was self-sufficient and able to walk ≥500 meters without aid or rest [11]. In addition, to assess the degree of disability, the EDSS score was further quantified on-site by an experienced neurologist, prior to the start of CDT, in patients randomized to the intervention; and in those control patients who chose to receive CDT after study completion.

The enrolment started February 2013 and ended April 2016. The follow-up started April 2013 (first 1-month assessment) and ended December 2016 (last 6-month assessment).

Patients and support partners gave their written informed consent. CDT was given during three consecutive days in Zorghotel Spelderholt, Beekbergen, the Netherlands, a facility especially equipped for the accommodation of people with impaired health. The number of sessions, their schedule, and their duration are presented in the Appendix (see App 1).

### Randomization

Patients were randomized in a 1:1 ratio to CDT or control group via stratified block randomization with disease duration and gender as blocking factors using block sizes of four. Patients randomized into the control group received any care or treatments that were deemed necessary by caregivers, and were given the opportunity to receive CDT after study completion of study participation. The sequence with which participants were allocated was generated at the Department for Health Evidence, Radboud university medical centre, Nijmegen, the Netherlands (RD). Patients were enrolled by the NMSF (MH). An independent statistician who had no involvement in the rest of the trial assigned the patients to the trial groups. Participants, the multidisciplinary team, those assessing the outcomes, and those analysing the data were not masked to group assignment.

### Outcomes and assessments

#### Primary outcome

The primary study outcome was the change in control self-efficacy, assessed by the Multiple Sclerosis Self-Efficacy Scale (MSSES), at six months [12]. The MSSES is a specific and sensitive self-report questionnaire consisting of two 9-items subscales for control and functional self-efficacy, with a high internal consistency and test-retest reliability [12]. Each item is scored from 10 (very uncertain) to 100 (very certain), and addition of the item scores yields the MSSES Control (MSSES-C) and Function (MSSES-F) scores (minimum 90, maximum 900). The MSSES-C measures confidence with managing symptoms and coping with the demands of illness, and the MSSES-F measures confidence with regard to functional abilities [12].

#### Secondary outcomes

Secondary endpoints were changes in functional self-efficacy (MSSES-F), participation and autonomy, HRQoL, anxiety, depression and coping styles at six months.

Participation and autonomy were assessed by the Impact on Participation and Autonomy (IPA) questionnaire [13]. The IPA-Limitations subscale assesses perceived limitations in participation and autonomy, and a higher score indicates higher limitations to participation and autonomy. The IPA-Problems subscale examines the extent to which these limitations are experienced as problematic, and a higher IPA-Problems score indicates a greater experience of problems [13].

HRQoL was assessed via the Multiple Sclerosis Quality of Life 54-Items (MSQoL-54) [14].

The MSQoL-54 is an MS-specific, multi-dimensional inventory of patient-centered health status, and consists of the Short Form 36-Items health survey as a generic core measure, supplemented with 18 questions on items relevant to patients with MS. A physical and a mental dimension underlie the MSQoL-54: the physical and mental domains [14]. Scores for each domain range from 0–100, where higher values indicate better HRQoL.

Anxiety and depression were measured by the Hospital Anxiety and Depression Scale (HADS), which is a 14-item, self-report questionnaire consisting of two subscales for anxiety and depression [15]. A total subscale score of eight to 10 points indicates possible mild to moderate symptoms of anxiety/depression, and 11 to 21 points indicate a probable clinically significant condition of anxiety/depression [15].

Coping styles were assessed with the Utrecht Coping List (UCL) [16]. The scores on the subscales ‘palliative reaction’ and ‘avoidance’ range from eight to 32, the scores on the subscales ‘active tackling’ and ‘passive reaction’ range from seven to 28, and the scores on the subscales ‘emotion expression’, ‘comforting cognitions’ and ‘social support’ range from three to 12, five to 20 and from six to 24, respectively.

#### Tertiary outcome

The tertiary endpoint was change in care-related strain on support partners at six months, which was assessed by the Caregiver Strain Index (CSI) [17]. The CSI includes thirteen items yielding a total score between zero and 13. A higher score indicates higher care-related strain, and scores of seven or higher are associated with caregivers' self-reports of experiencing situations that conflict with giving help [17].

#### Assessment schedule

Assessments were at baseline and at one, three and six months follow-up via online questionnaires (www.ms4ri.nl). Data were acquired with use of the open source online application LimeSurvey® in compliance with European Union regulations concerning online medical data. For patients the completion of the questionnaires took about 45 minutes and for partners about five minutes per time point.

#### Safety and adverse events assessment

During CDT, safety aspects and adverse events were assessed and documented by the multidisciplinary team at the end of each day. After having completed CDT, participants were informed to contact the help desk of the NMSF or the psychiatrist of the team (RPR) in case of questions or complaints.

### Statistical aspects

The sample size calculation was based on the difference in MSSES-C score between baseline and six months in low-disability RRMS patients in the observational study [9]. We assumed at six months an average MSSES-C score of 650 for the CDT group and of 560 for the control group. Assuming a standard deviation (SD) of 200 at the 6-month measurement, 79 patients were needed per group to obtain a power of 80%, using a two-sided *t*-test with an alpha of 5%. However, as we used the baseline measurement in the analysis, this number had to be multiplied by a design factor equalling 1 –r^2^ baseline, 6 months. Assuming a correlation of 0.5, based on the observational data, this design factor equals 0.75. Therefore, the number of analyzable patients needed to obtain a power of 80% equalled approximately 60 per group. Taking into account an expected drop-out of at least 15% we aimed to include a minimum of 140 patients.

All endpoints were analyzed according to the intention-to-treat principle using an ANCOVA with the 6-month value as dependent variable, and condition, baseline value, disease duration and gender as independent variables. Hedges’ *g* was used to calculate the effect size for the primary outcome in order to understand the effect of the intervention.

Post hoc analyses included ANCOVA with the 1-month and 3-month values as dependent variables, and condition, baseline values, disease duration and gender as independent variables; multiple regression analysis to detect the contribution of age, gender, disease duration, EDSS score and education at six months, and using graphical inspection of the residuals to check whether the assumptions of the analysis model were fulfilled; related-samples Wilcoxon signed rank tests for within-group comparisons of follow-up versus baseline; and *Χ*^2^-tests to compare ‘early permanent responders’ (i.e. improved control self-efficacy at both one and six months), ‘delayed responders’ (i.e. improvement at six months but not at one month), and ‘early non-permanent responders’ (i.e. improvement at one but not at six months), whereby improvement was defined as an increase of at least 75 points compared to baseline, being ≥0.5 SD baseline (n = 155). Moreover, at each follow-up time point we calculated for every patient the percentage change in MSSES-C from baseline, added the respective percentages, and divided the sum by the number of observations, resulting in a mean percentage change from baseline per time point.

The analyses were performed at the Department of Health Services Research, Care and Public Health Research Institute, Maastricht University, Maastricht, the Netherlands (GAvM) and the Department for Health Evidence, Radboud university medical centre, Nijmegen, the Netherlands (RD). The statistics program used was Statistical Package for the Social Sciences (IBM Corporation, Armank, U.S.A.) for Windows version 24. In all analyses p-values < 0.05 were considered statistically significant. Analyses beyond the primary outcome were exploratory, and therefore the type I error rate was not strictly controlled.

## Results

**Fig 1:**
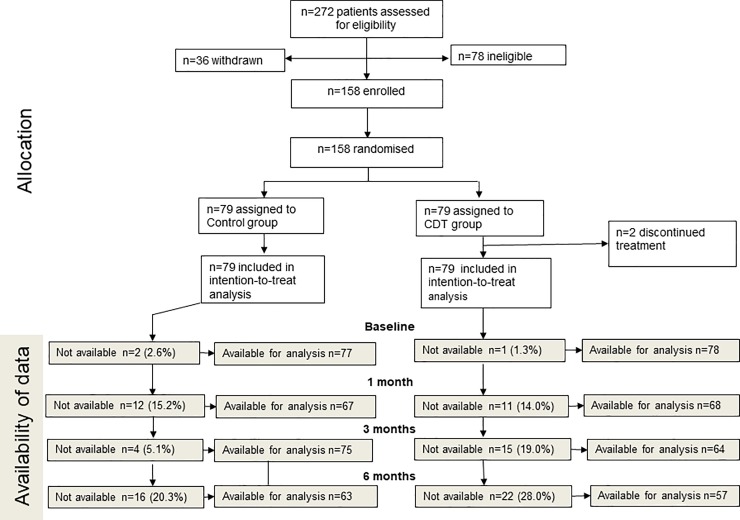
Patient flow diagram.

A total of 272 patients were screened for eligibility (Fig 1). Of the 194 eligible patients, 36 decided not to participate, 79 were assigned to CDT, and 79 to the control group (Fig 1). The full analysis set (intention-to-treat population) for the primary outcome comprised 158 patients.

The baseline demographic and disease characteristics of the intention-to-treat population are presented in Table 1.

**Table 1:** Demographic and disease characteristics of the intention-to-treat population.

	CDT (n = 79)	control (n = 79)
**Age, years**		(n = 77)
Mean (SD)	40 (8.7)	40 (9.4)
Median (range)	41 (20–61)	40 (23–61)
**Gender, n (%)**		
Women	69 (87.3%)	70 (88.6%)
Men	10 (12.7%)	9 (11.7%)
**Living situation, n (%)**	(n = 75)	(n = 73)
Alone	16 (23.0%)	15 (19.0%)
With one or more people	59 (74.6%)	58 (73.3%)
Marital status, n (%)	(n = 75)	(n = 73)
Living together, married	50 (66.3%)	44 (55.7%)
Single	19 (22.4%)	18 (22.8%)
Divorced	5 (6.3%)	7 (8.9%)
Other	1 (1.3%)	4 (5.1%)
**Education, n (%)**	(n = 75)	(n = 73)
Primary school or lower education	2 (2.5%)	3 (3.8%)
Middle education	26 (33.0%)	31 (39.3%)
Higher education	47 (59.5%)	39 (49.4%)
**Work status, n (%)**		
Employed, including self-employed	26 (32.9%)	37 (46.9%)
Disabled	35 (44.3%)	34 (43.0%)
Sick leave	19 (24.1%)	18 (22.8%)
Voluntary work	21 (26.6%)	16 (12.3%)
Applicant for job	17 (21.5%)	5 (6.3%)
Other (student, retired)	11 (14.0%)	4 (5.1%)
**EDSS**	(n = 78)	(n = 52)
Mean (SD)	2.3 (1.03)	2.3 (1.13)*
Median (range)	2.5 (0–4)	2.5 (0–4.5)
**Time since diagnosis, years**		(n = 78)
Mean (SD)	6.5 (5.6)	6.5 (5.3)
Median (range)	5 (0–25)	5 (1–21)
**Self-reported health disorders, n (%)**		
No	69 (87.3%)	64 (81.0%)
Yes	6 (7.6%)	9 (11.4%)

CDT: Can do treatment; SD: standard deviation; EDSS: Expanded Disability Status Scale; *, assessed at six months.

CDTs were given between April 5, 2013 and May 29, 2016. In the CDT group 61 (77.2%) patients participated with support partner, and in the control group 47 (59.5%). The intervention was delivered as planned in all patients except for two (2.5%) patients, who discontinued CDT prematurely. Follow-up data at 1, 3 and 6 months were not available for 11 (14%), 15 (19%) and 22 (28%) CDT patients, and for 12 (15%), 4 (5%) and 16 (20%) control patients, respectively (Fig 1).

### Pre-specified analyses

**Fig 2:**
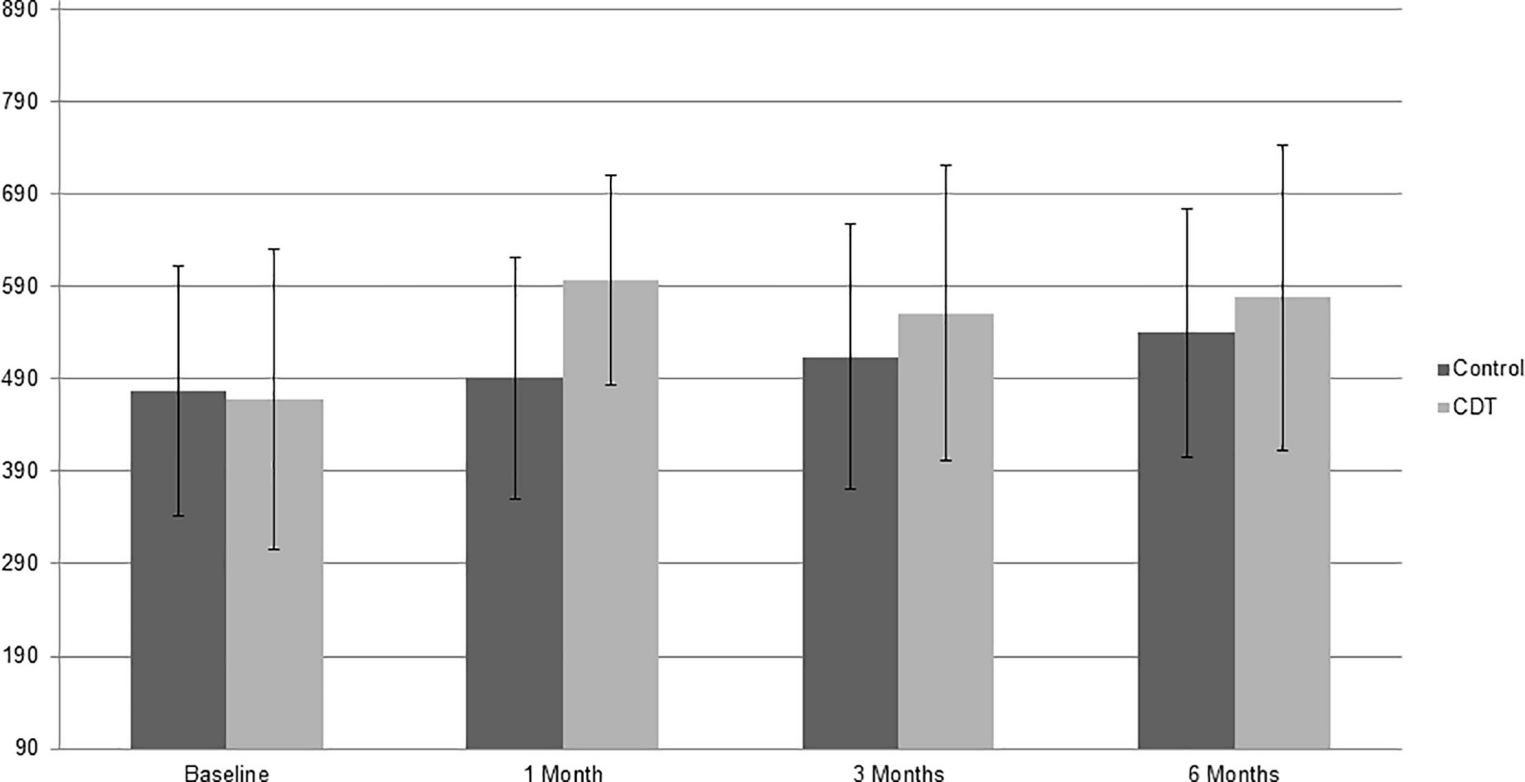
Mean (bars) and SD (whiskers) values of MSSES Control scores (minimum 90, maximum 900) at baseline and at one, three and six months follow-up in CDT and control groups. SD: standard deviation; MSSES: Multiple Sclerosis Self-Efficacy Scale; CDT: can do treatment.

The baseline and follow-up values for the primary, secondary and tertiary outcomes in both groups, and the between-group differences at follow-up are presented in Table 2. At six months the MSSES-C score did not statistically significantly differ between the two groups. The mean (SD) score in the CDT group was 578 (166) and in the control group 540 (135) (Fig 2, Table 2), and the mean (95%Confidence Interval [CI]) between-group difference was 18 (-8, 88) (p = 0.100). The MSSES-C effect size at six months was .25, indicating a small effect of CDT.

**Table 2:** Baseline and follow-up outcomes in the CDT (n = 79) and control (n = 79) groups, and between-group differences at follow-up.

	Baseline		One month				Three months				Six months			
	Control	CDT	Control	CDT	Difference		Control	CDT	Difference		Control	CDT	Difference	
	Mean (SD)	Mean (SD)	Mean (SD)	Mean (SD)	p*	95% CI	Mean (SD)	Mean (SD)	p*	95% CI	Mean (SD)	Mean (SD)	p*	95% CI
**MSSES-Control**	477 (136)	468 (162)	491 (131)	597 (114)	.000	[75,146]	514 (143)	561 (160)	.018	[10,100]	540 (135)	578 (166)	.100	[–8,88]
**MSSES0-Function**	701 (93)	693 (113)	691 (95)	724 (89)	.002	[14,64]	697 (107)	706 (137)	.582	[–28,49]	711 (79)	703 (116)	.765	[–38,28]
**IP Limitations**	1.5 (0.6)	1.6 (0.6)	1.5 (0.6)	1.3 (0.6)	.000	[-.4,-0.2]	1.5 (0.7)	1.4 (0.7)	.011	[-0.4,-0.0]	1.4 (0.7)	1.3 (0.7)	.329	[-0.3,1.0]
**IPA Problems**	1.0 (0.5)	1.1 (0.5)	1.1 (0.5)	1.0 (0.5)	.103	[-.2,0.2]	1.1 (0.5)	1.0 (0.6)	.048	[-0.3,-0.0]	1.0 (0.5)	0.9 (0.6)	.118	[-0.3,0.0]
**MSQoL-54 Mental**	63.4 (20.1)	60.9 (17.6)	64.1 (19.2)	69.1 (15.7)	.007	[1.6,10.2]	66.2 (20.2)	67.0 (16.2)	.192	[-1.6,7.9]	67.3 (18.2)	65.9 (17.8)	.719	[-6.4,4.4]
**MSQoL-54 Physical**	53.2 (13.6)	54.7 (15.3)	55.3 (15.1)	64.1 (14.6)	.000	[3.9,11.0]	56.4 (14.3)	60.7 (17.1)	.099	[-0.7,7.5]	58.1 (14.0)	59.2 (17.2)	.510	[-6.1,3.1]
**HADS Anxiety**	7.7 (4.4)	8.4 (4.0)	7.5 (4.4)	6.5 (3.9)	.000	[-2.5,-0.7]	6.7 (4.1)	6.8 (4.1)	.370	[-1.5,0.6]	6.6 (4.3)	6.5 (4.3)	.506	[-1.4,0.7]
**HADS Depression**	6.3 (4.2)	6.4 (3.6)	6.3 (4.1)	5.0 (3.8)	.008	[-2.1,-0.3]	5.9 (4.5)	5.3 (3.8)	.131	[-1.8,0.2]	5.1 (4.0)	5.2 (4.1)	.623	[-0.8,1.2]
**UCL Active tackling**	18.3 (4.2)	17.4 (4.3)	17.5 (3.9)	18.9 (3.8)	.000	[1.1,2.8]	18.5 (4.3)	18.6 (3.8)	.101	[-0.1,1.5]	18.4 (4.2)	18.7 (4.0)	.277	[-0.5,1.6]
**UCL Palliative**	18.3 (3.6)	17.6 (3.6)	17.6 (3.8)	17.6 (3.5)	.032	[0.1,1.8]	17.9 (3.6)	17.9 (3.8)	.078	[-0.1,1.8]	17.7 (3.8)	17.8 (3.7)	.039	[0.1,2.0]
**UCL Avoidance**	17.2 (3.7)	17.0 (3.4)	16.9 (3.7)	16.8 (3.3)	.925	[-0.8,0.9]	16.2 (3.4)	17.1 (3.3)	.054	[-0.0,1.9]	16.8 (3.3)	17.4 (3.3)	.054	[-0.0,2.0]
**UCL Social support**	13.5 (4.3)	13.2 (4.2)	13.1 (4.3)	13.7 (3.8)	.003	[0.5,2.1]	13.9 (4.2)	14.0 (3.9)	.293	[-0.4,1.4]	13.6 (4.6)	13.6 (4.0)	.923	[-1.0,1.1]
**UCL Passive**	13.0 (3.5)	13.2 (3.2)	12.9 (3.7)	11.9 (3.3)	.006	[-1.8,-0.3]	12.3 (3.8)	12.3 (3.2)	.562	[-1.0,0.5]	12.2 (3.9)	12.6 (4.0)	.275	[-0.4,1.5]
**UCL Emotion**	6.0 (1.7)	6.4 (1.9)	6.1 (1.7)	5.9 (1.4)	.097	[-0.8,0.1]	5.9 (1.7)	6.0 (1.9)	.825	[-0.4,0.5]	6.1 (1.8)	5.9 (1.5)	.538	[-0.6,0.3]
**UCL Comforting**	12.6 (2.7)	12.7 (2.9)	12.5 (2.6)	12.7 (2.6)	.608	[-0.5,0.9]	12.3 (2.6)	12.4 (2.9)	.728	[-0.6,0.9]	12.4 (2.7)	12.5 (3.0)	.782	[-0.7,0.9]
CSI**	4.6 (2.8)	5.1 (3.0)	4.6 (3.1)	4.4 (2.6)	.176	[-1.3,0.2]	4.7 (2.9)	4.4 (2.8)	.182	[-1.3,0.3]	4.7 (3.0)	5.0 (3.2)	.724	[-1.2,0.8]

CDT: can do treatment; MSSES: Multiple Sclerosis Self-Efficacy Scale; SD: standard deviation; IPA: Impact on Participation and Autonomy; MSQoL-54: Multiple Sclerosis Quality of Life-54 Items questionnaire; HADS: Hospital Anxiety and Depression Scale; UCL: Utrecht Coping List; CSI: Caregiver Strain Index

*ANCOVA (see Methods)

** partners of MS patients.

As to the secondary and tertiary endpoints, at six months the UCL palliative reaction score was slightly higher in the CDT group ([0.1,2.0], p = 0.039), while the other outcomes showed no between-group differences (Table 2).

### Post hoc analyses

Multiple regression analysis with age, gender, disease duration, EDSS score and education as independent variables and MSSES-C at six months as a dependent variable showed no associations. At six months, 31 of 54 (57.4%) CDT patients and 26 of 56 (46.4%) control patients had an improved control self-efficacy, whereby improvement was defined as an increase of at least 75 points compared to baseline, being ≥0.5 SD baseline (n = 155). ANCOVA analyses showed that at one and three months the mean (95%CI) between-group difference in MSSES-C score was 108 (75,146) (p = .000) and 45 (10,100) (p = .018), respectively, in favour of the CDT group (Table 2). As to within-group comparisons, at all three follow-up time points, and in both the CDT and the control group, the MSSES-C score was higher than at baseline (all p-values >.001). Actually, at one, three and six months the mean (SD) change in MSSES-C score from baseline was +54.0% (92.7%), +38.5% (89.1%) and +42.6% (91.4%) in the CDT group and +10.4% (41.8%), +13.8% (45.9%) and +24.8% (47.9%) in the control group. In the CDT group 29 of 54 (53.7%) patients of whom the one and six month values were available, were early ‘permanent responders’, and in the control group 11 of 56 (19.6%) patients (p = 0.000; *Χ*^2^-test). The frequency of ‘delayed responders’ (n = 3 in CDT vs. n = 15 in control) and of ‘early non-permanent responders’ (n = 4 in CDT vs. n = 4 in control) did not differ between the groups (both p = 0.077, *Χ*^2^-test).

As to the secondary and tertiary endpoints, at three months the IPA Limitations score was lower in the CDT group than in the control group (p = 0.011) (Table 2), and at one month the MSSES-F, IPA Limitations, HADS Anxiety and Depression, mental and physical MSQoL-54, and UCL active tackling, palliative reaction, social support, and passive reaction scores favoured the CDT group (Table 2).

### Safety and adverse events

No safety concerns or adverse events were noted during the intervention. Moreover, the follow-up HADS data did not suggest that an increase in depressive symptoms or anxiety occurred more frequently in patients in the CDT group than in the control group (Table 2). However, the two patients who had prematurely discontinued CDT, contacted the NMSF after the intervention with complaints: one patient (female, 44 years, disease duration 24 years) stated that the treatment did not meet her expectations, and one patient (female, 38 years, disease duration 10 years) stated that the psychiatrist was too confronting and that the treatment was damaging.

## Discussion

### Principal findings

This RCT investigated the effect of CDT, an intensive 3-day multidisciplinary social cognitive theory-based intervention, on control self-efficacy in low-disability RRMS patients. The MSSES-C score at six months—the trial’s primary endpoint—did not differ between the intervention and control groups. However, at one and three months the MSSES-C score was higher in CDT than control patients.

### Findings in context

Only few RCTs investigating the effect of social cognitive approaches on self-efficacy in RRMS have been reported. Three RCTs in US American patients on physical activity were negative with respect to exercise self-efficacy—which was in all studies a secondary outcome [5–7]. Recently, a RCT in 91 MS patients– 86% RRMS and the majority experiencing mild to moderate disability—found that a 12-wk educational socialization program resulted in improved self-efficacy compared with control group participants [8]. Notably, various features distinguish our study from these RCTs, such as the role of a patient organisation in study initiation, funding, patient recruitment and data collection; the direct-to-patient recruitment procedure; and the large, representative sample size.

Despite an evident improvement of control self-efficacy in the CDT group at six months, the difference with the controls was not statistically significant. Interestingly, we observed an early improvement (one month) after CDT, but not in the controls; and, while the improvement in the CDT group was largely maintained at six months, the control group improved gradually over time (Fig 2). The change in the control patients was unexpected, for, in a 30-month prospective longitudinal study in RRMS, exercise self-efficacy did not change throughout the whole study period [18]; and a recent study in twins, investigating the causal structure of general self-efficacy in young people, found that 75% of variation in self-efficacy was due to genetic factors [19].

The improvement in the control group could be related to various factors. First, we used a waitlist control, and 61 (77%) of the control patients did indeed chose to receive CDT after the study. It has been known that waitlist control groups may behave in different and unpredictable ways. Thus, an exploratory RCT evaluating the impact of a waitlist control design, reported that in persons with problem drinking–a chronic health condition–the outcomes had improved by 25–35% after four weeks in the waitlist group [20]. So, in our controls the prospect of eventually receiving CDT may have elicited positive expectations that led to the anticipation of the desired effect. Second, study procedures could have played a role, like the repeated completion of questionnaires or the personal contact during the eligibility assessment by phone [21]. Third, people build their self-efficacy by observing successful efforts made by others and by verbal persuasion that one is capable of managing one’s challenges [1]. During the 3-year period from first-patient-in to last-patient-out, control patients may have communicated with participants who reportedly benefited from CDT, and may thus have become convinced of CDT’s impact. Importantly, the increased use of social media in recent years is likely to have facilitated such communication. Moreover, to promote recruitment, the NMSF actively communicated the potential benefits of CDT via folders and in the media. Fourth, patients were free to receive outside of the study any care that was deemed necessary, and most patients did receive at least some psychological, psychotherapeutic, psychiatric or social-psychiatric nursing care; however, the amount of care was low, and there were no differences between control and CDT patients (data not shown).

An economic evaluation was attached to this RCT, which assessed from a societal perspective the cost-effectiveness of the intervention [22]. When using the EuroQol-5 Dimensions-5 Levels questionnaire to calculate a quality-adjusted-life-year, CDT was not cost-effective. However, when using the MSSES-C or Short Form-6 Dimensions (part of the MSQoL-54) as outcomes, there is a probability that CDT is cost-effective [22].

### Strengths and limitations

The study has several strengths, such as the large sample size of 158 patients, and the recruitment of patients from all over the Netherlands. Moreover, the number of patients who were not treated according to the protocol was low, and the analysis of the data was performed by independent researchers who were not otherwise involved in the trial. Importantly, the follow-up was longer than in other RCTs on self-efficacy. E.g. in a recent report by Kalina et al. on the positive effect of a 12-week educational socialization programme on self-efficacy in MS, the post-treatment assessment was scheduled immediately after the programme’s completion [8].

On the other hand, the study has several limitations. First, the follow up may have been too short to assess the full effects of CDT, as a recent observational study showed improved anxiety and depression 12 months after the intervention, in addition to improved control self-efficacy [23]. Second, the direct-to-patient design and the role of the patient organization may have led to biased or partial information or unwanted interactions between participants. Third, while in the observational study all patients participated with a support partner, in this study about one third of the patients were unaccompanied, which might have interfered with the full development of the CDT’s effects.

## Conclusions

An unmasked RCT assessed the effect of CDT, an intensive 3-day multidisciplinary social cognitive theory-based treatment, on control self-efficacy in low-disability RRMS patients and found no difference compared with waitlist controls who had the option to receive the intervention after study participation.We think that the CDT concept may be further developed, e.g. by selecting patients with evident points of interaction, such as stressors, high levels of distress or relational problems with (support) partner [24], and by applying out-patients settings or web-based applications.

## Supporting information

S1 File Appendix. (DOCX)

S2 File Minimal anonymized data set STATA. (DTA)

S3 File Minimal anonymized data set SPSSS. (SAV)

S4 File CONSORT checklist. (DOCX)

## Abbreviations

CDT: Can do treatment
CSI: Caregiver Strain Index
EDSS: Expanded Disability Status Scale
HADS: Hospital Anxiety and Depression Scale
HRQoL: Health-Related Quality of Life
IPA: Impact on Participation and Autonomy
MSSES: Multiple Sclerosis Self-Efficacy Scale
RRMS: Relapsing Remitting Multiple Sclerosis
MS: Multiple Sclerosis
MSQoL-54: Multiple Sclerosis Quality of Life-54 Items
SD: Standard deviation
RCT: Randomised controlled trial
UCL: Utrecht Coping List
95% CI: 95 percent Confidence Interval
