# Supplementary material for: Effect of an intensive 3-day social cognitive treatment (can do treatment) on control self-efficacy in patients with relapsing remitting multiple sclerosis and low disability: A single-centre randomized controlled trial
Source: PLoS One. 2019 Oct 10;14(10):e0223482. doi: 10.1371/journal.pone.0223482 (PMC6786633; doi:10.1371/journal.pone.0223482)
Supplement: S1 File — (DOCX) [file pone.0223482.s001.docx]

APPENDIX

PROGRAM Can do treatment

| **Day 1** | | |
| --- | --- | --- |
| **TIME** | **DESCRIPTION** | **ROOM** |
| 10.30 | Presence Medical Team | VIJVERZICHT |
| 10.00-11.00 | Welcom of participants | BAR |
| 11.00-11.15 | Information about the study by Dr. Jongen | VIJVERZICHT |
| 11.15-11.30 | "Why Are You Here?"- information about the content of the Can Do Weekend | VIJVERZICHT |
| 11.30-12.30 | “Can Do – what is that?”- discussion of program and objectives | VIJVERZICHT |
| 12.30-13.30 | LUNCH | RESTAURANT |
| 13.30-15.00 | Plenary conversation | VIJVERZICHT |
| 15.00-15.30 | PAUSE | BAR |
| 15.30-17.00 | Group 1: description of personal goals | VIJVERZICHT |
| 15.30-17.00 | Groep 2: relaxation exercises | BOSZICHT |
| 17.00-17.30 | PAUSE | BAR |
| 17.30-19.00 | Group 1: relaxation exercises | BOSZICHT |
| 17.30-19.00 | Groep 2: description of personal goals | VIJVERZICHT |
| 19.00-20.30 | Dinner | RESTAURANT |
| 20.30- | Preparations for second (theatre) evening | N.A. |
| 20.30-21.30 | Tip time | VIJVERZICHT |
| **Day 2** | | |
| **TIME** | **DESCRIPTION** | **ROOM** |
| 07.30-08.00 | Walk in the surroundings | N.A. |
| 08.00-09.00 | Breakfast | RESTAURANT |
| 09.00-10.30 | Plenary conversation | VIJVERZICHT |
| 10.30-11.00 | PAUSE | BAR |
| 11.00-12.30 | CONSULATIONS CAROUSEL | VIJVERZICHT, BOSZICHT, FITNESS AND SITTING ROOM |
| 12.30-13.30 | LUNCH | RESTAURANT |
| 13.30-15.00 | Small Group Sessions 1 (LIFE, FEELING or BODY) | VIJVERZICHT, BOSZICHT AND FITNESS |
| 15.00-15.30 | PAUSE | BAR |
| 15.30-17.00 | Small Group Sessions 2 (LIFE or FEELING) | VIJVERZICHT AND BOSZICHT |
| 17.00-17.30 PAUSE BAR | | |
|  | | |

| 17.30-19.00 | Relaxation exercises –Yoga/ relaxation by effort –Fysio | FITNESS AND BOSZICHT |
| --- | --- | --- |
| 19.00-20.30 | Diner | RESTAURANT |
| 20.00-21.15 | Tip time | VIJVERZICHT |
| 20.30-21.30 | Preparation of evening program | THEATRE |
| 21.30-22.30 | Presentations etc. by participants/partners (+ CART W BEVERAGES) | THEATRE |
| **Day 3** | | |
| **TIME** | **DESCRIPTION** | **ROOM** |
| 07.30-08.00 | Walk in surroundings | N.A. |
| 08.00-09.00 | Breakfast | RESTAURANT |
| 09.00-09.30 | Plenary conversation | VIJVERZICHT |
| 09.30-10.45 | Small Group Sessions 3 (LIFE, FEELING or BODY) | VIJVERZICHT, BOSZICHT AND FITNESS |
| 10.45-11.15 | PAUSE | BAR |
| 11.15-12.30 | Small Group Sessions 4 (LIFE, FEELING or BODY) | VIJVERZICHT, BOSZICHT AND FITNESS |
| 12.30-13.30 | LUNCH | BAR |
| 13.30-14.45 | Small Group Sessions 5 (LIFE, FEELING or BODY) | VIJVERZICHT, BOSZICHT AND FITNESS |
| 14.45-15.15 | PAUSE | BAR |
| 15.15-16.45 | Plenary & round off Can Do Weekend/ Collecting final info, say goodbye | VIJVERZICHT |
| 16.45-17.15 | Say goodbye to participants | ENTREE |
| 17.15-18.30 | Final evaluation by team | VIJVERZICHT |
| 18.30-19.30 | Diner team (PLATE SERVICE) | RESTAURANT |
